# Protective Effect of Selected Medicinal Plants against Hydrogen Peroxide Induced Oxidative Damage on Biological Substrates

**DOI:** 10.1155/2014/861084

**Published:** 2014-11-12

**Authors:** Namratha Pai Kotebagilu, Vanitha Reddy Palvai, Asna Urooj

**Affiliations:** Department of Studies in Food Science and Nutrition, University of Mysore, Manasagangothri, Mysore 570006, India

## Abstract

Oxidative stress is developed due to susceptibility of biological substrates to oxidation by generation of free radicals. In degenerative diseases, oxidative stress level can be reduced by antioxidants which neutralize free radicals. Primary objective of this work was to screen four medicinal plants, namely, *Andrographis paniculata*, *Costus speciosus*, *Canthium parviflorum*, and *Abrus precatorius*, for their antioxidant property using two biological substrates—RBC and microsomes. The antioxidative ability of three solvent extracts, methanol (100% and 80%) and aqueous leaf extracts, was studied at different concentrations by thiobarbituric acid reactive substances method using Fenton's reagent to induce oxidation in the substrates. The polyphenol and flavonoid content were analyzed to relate with the observed antioxidant effect of the extracts. The phytochemical screening indicated the presence of flavonoids, polyphenols, tannins, and *β*-carotene in the samples. In microsomes, 80% methanol extract of *Canthium* and *Costus* and, in RBC, 80% methanol extract of *Costus* showed highest inhibition of oxidation and correlated well with the polyphenol and flavonoid content. From the results it can be concluded that antioxidants from medicinal plants are capable of inhibiting oxidation in biological systems, suggesting scope for their use as nutraceuticals.

## 1. Introduction

It is well known that oxidant by-products of normal metabolism such as free radicals and reactive oxygen species (ROS) in excess can cause extensive damage to DNA, proteins, and lipids [1]. Under stress, the body produces more ROS, such as superoxide anion and hydroxyl radical, which are highly reactive and potentially damaging transient chemical species. These are continuously produced in the human body, as they are essential for energy supply, detoxification, chemical signaling, and immune function. Overproduction of free radicals and ROS induced by exposure to external oxidant substances or a failure in the defense mechanisms causes oxidative stress in turn leading to various degenerative diseases of aging such as cancer, cardiovascular disease, cataracts, immune system decline, and brain dysfunction [2, 3].

Antioxidants reduce the oxidative stress in cells and are therefore useful in the treatment of many human diseases. There are various endogenous and exogenous sources of antioxidants. The endogenous sources include antioxidant enzymes such as superoxide dismutase, catalase, glutathione peroxidase, and low-molecular weight antioxidants and exogenous sources such as food sources and medicinal plants.

Several* in vitro*,* ex vivo*, and* in vivo* methods are used to measure the antioxidant potential of plants and foods. It is well known that the antioxidant capacity of a sample will differ with the different oxidants it is measured against, the substrate used (food or biological substrate), and the analytical method used to measure the overall antioxidant capacity. It is also important to examine the effect of antioxidant on markers of oxidative stress. Antioxidant activity can be indirectly assessed by monitoring levels of oxidative stress. Several biomarkers can be used to assess oxidative damage to lipids, protein, and DNA. Thiobarbituric acid reactive substances (TBARS) method is used to measure the level of resistance to oxidation. It determines the resistance of lipids and proteins to oxidation in the presence of an antioxidant. The malondialdehyde (MDA) generated through oxidative degradation of polyunsaturated fatty acids forms a complex with thiobarbituric acid (MDA-TBA complex) to give a pink color which can be measured spectrophotometrically [4]. These peroxidation products are measured to monitor levels of oxidative stress and are thus an indirect measure of antioxidant capacity.

Biological substrates such as brain, microsomes, low density lipoprotein (LDL), red blood cells (RBC), and cholesterol are susceptible to oxidation due to their composition. Microsomes and RBC are composed of proteins which on oxidation yield protein by-products. Denatured haemoglobin products, particularly free heme, can promote membrane damage and hemolysis through mechanisms including lipid peroxidation and oxidation of protein thiol groups [5]. The disruption of normal processes through injury or the presence of a xenobiotic may greatly enhance the microsomal source of free radicals, resulting in chemical modifications of surrounding structures [6].

Extracts from the stems, roots, bark, leaves, fruits, and seeds of many plants have antioxidant potential. Natural antioxidants represent a potentially side-effect-free alternative to synthetic antioxidants [2]. Phytomedicines are active ingredients exclusively derived from plants which are capable of preventing, curing, or managing a disease. Various medicinal plants have been studied and used for their capability in curing disease.

Literature reports the use of various parts of medicinal plants from the Western Ghats of Southern India in the treatment of several diseases. The whole plant of* Abutilon indicum* has been used in urinary diseases and inflammation, the leaves of* Alstonia scholaris* have been used in the treatment of asthma, beriberi, ulcers, and tumors, and the fruit of* Catunaregam spinosa* has been used in the treatment of diarrhea and dysentery while the root of* Asparagus racemosus* is reported to be beneficial in the treatment of piles, gastritis, cough, and diarrhea [7].

In our laboratory, biscuits prepared by incorporating the extracts of Amla powder (*Emblica officinalis*), drumstick leaves (*Moringa oleifera*), and raisins (*Vitis vinifera*) exhibited better shelf life and were acceptable in sensory characteristics [8]. Other medicinal plants, namely,* Morus indica* [9],* Moringa oleifera*,* Aegle marmelos* [10],* Raphanus sativus*, olive leaves [11], pomegranate peel [11],* Costus speciosus* [12],* Canthium parviflorum* [12], and* Abrus precatorius* [13] leaf extracts, have effectively inhibited the rate of oxidation in the edible oil emulsions.

The selected medicinal plants for the study,* Andrographis paniculata*,* Costus speciosus*,* Canthium parviflorum*, and* Abrus precatorius*, have been reported to exhibit antioxidant, antipyretic, antibacterial, antiviral, anticancer, hypoglycemic, hepatoprotective, gastroprotective, immunoprotective, and cardioprotective effect [14–19]. These four plants were selected to investigate their antioxidative ability in biological substrates based on our earlier investigations which revealed their ability to inhibit oxidation in food system [12, 13]. In this* ex vivo* study, the antioxidative ability of various solvent extracts of these four plants to prevent oxidative damage against Fenton's reagent induced peroxidation in human erythrocytes and rat liver microsomes were explored.

## 2. Materials and Methods

### 2.1. Chemicals

All the chemicals used were of analytical grade. Thiobarbituric acid, triethanolamine, rutin, dithiothreitol, and EDTA were purchased from Hi Media Pvt. Ltd., Mumbai. DTNB was purchased from Sigma chemicals. Protein kit was purchased from Span Diagnostics Ltd., Gujarat.

### 2.2. Plant Materials

The plant samples selected for the study were* Andrographis paniculata* (AnP),* Costus speciosus* (CS),* Canthium parviflorum* (CP), and* Abrus precatorius* (AP) which were collected from the Western Ghats. The plant samples were identified by Dr. Janardhan, Department of Studies in Botany, University of Mysore, and voucher specimen was retained in the laboratory for future reference. The leaves were cleaned, washed, and dried in hot air oven at 55°C for 8–10 h. The dried leaves were ground to a fine powder and passed through 60 sieve mesh and stored in air tight containers until use.

### 2.3. Preparation of Extracts

Three different extracts were prepared from the dehydrated samples, that is, 100% methanol (ME), 80% methanol (methanol 80 : water 20) (80 ME), and 100% aqueous (AQ). 10 g of each sample was extracted in 100 mL of the solvent in a mechanical shaker for 12 hours and filtered with Whatman number 1 filter paper. The filtrate obtained from ME and 80 ME was evaporated to dryness in a rotary evaporator at 50°C (Superfit, Bangalore) and aqueous extract was freeze dried (Thermo Electron Corporation, Pune). The extracts were stored at 4°C until further use.

### 2.4. Estimation of Phytochemicals

The samples were analyzed for various antioxidant components using standard methods. Ascorbic acid was determined according to the titrimetric method using 2, 6-dichlorophenol-indophenol dye [20]. *α*-Tocopherol was extracted by direct saponification of dried sample and estimated based on formation of a red complex from reaction of *α*,*α*′-bipyridyl with ferrous ion due to reduction of ferric ion by tocopherol [21]. *β*-Carotene was quantified by open column chromatography, followed by measuring the absorbance of elute at 450 nm against standard *β*-carotene [20]. Reduced glutathione was determined based on the development of a yellow compound due to reaction of DTNB (5, 5-dithio(bis)nitrobenzoic acid) with compounds containing sulphydryl groups [22]. Total phenols were extracted from a weighed portion (1 g) of dried sample with 5 mL of 1.2 M HCl in 50% aqueous methanol for 2 h and with 70% acetone shaken for 2 h and analyzed by Folin-Ciocalteu micromethod [23]. Results were expressed as mg or g of gallic acid equivalent. The flavonoid content was determined by pharmacopoeia method [24] using rutin as a reference compound. Alkaloids were analyzed by spectrophotometric method using atropine as reference standard [25]. Saponins and steroidal saponins were estimated using diosgenin as standard [26, 27].

### 2.5. Isolation of Substrates

Two substrates, namely, RBC and microsomes, were chosen to study the potency of selected samples in inhibiting oxidation. Blood (20 mL) was drawn from healthy human volunteers, in vials, and centrifuged at 500 g for 10 min at 4°C and plasma was separated. RBC were washed with cell wash buffer in 1 : 2 ratio (RBC-1 : Buffer-2) and centrifuged at 500 g for 10 min at 4°C. The supernatant was removed and the procedure was followed 2-3 times [28]. The study was approved by the Human Ethics Committee of the University of Mysore (IHEC-UOM number 36 Res/2013-14, 16.04.2013).

Microsomes were isolated from the liver of healthy adult male rats from Central Animal House of the University of Mysore. Permission from Institutional Animal Ethics Committee of the University of Mysore was taken (UOM/IAEC/04/2013 dated 28-09-2013). The liver tissue was minced, homogenized, and centrifuged for 10 min at 12,000 g to remove cell debris and mitochondria. The supernatant solution was carefully removed to prevent contamination with mitochondria and recentrifuged for 10 min at 12,000 g to ensure removal of mitochondria; it was then centrifuged at 60,000 g for 60 min. The microsomal pellet was rinsed with buffer and frozen at −20°C. Frozen microsomes were resuspended in 0.1 M triethanolamine buffer, pH 7.4 containing 0.02 M EDTA, and 10 mM dithiothreitol. It was allowed to stand for 60 min packed in ice and diluted with buffer to give a protein concentration of 5–10 mg/mL [29].

### 2.6. Estimation of Thiobarbituric Acid Reactive Substances (TBARS)

Lipid peroxide formation was measured by the modified method of Ohkawa et al. [30]. Substrate (RBC and microsomes) equal to 1 mg protein was taken for the experiment. Plant extracts of different concentrations (300–500 *μ*L of 10 mg/mL concentration) were added to the substrates. Fenton's reagent was added to the substrates to induce oxidation (500 *μ*L of 20 mM FeSO_4_ and 250 *μ*L of 20 mM H_2_O_2_). In case of RBC, 1 mL trichloroacetic acid (TCA, 10%) was added to precipitate the RBC and both substrates were incubated for 2 h at 50°C. After 2 h, the RBC were separated by centrifuging at 3000 rpm for 10 min at 5°C. The supernatant was separated and 1 mL of thiobarbituric acid (TBA, 0.67%) was added. To the microsomes, 1 mL of TBA (0.67%) and 1 mL of TCA (10%) were added and kept in boiling water bath for 30 min and cooled. After cooling, 3 mL butanol was added to the tubes and vortex mixed. The pink color in butanol layer was measured at 532 nm [4]. IC_50_ values were calculated using mean values of triplicates of the inhibition of oxidation by the extracts at 300–500 *μ*L. Consider

(1) %  Inhibition  of  oxidation  =O.D  of  Control−O.D  of  sample[O.D  of  control]×100.

### 2.7. Statistical Analysis

All the experiments were carried out in triplicate (*n* = 3).

The correlation was determined by Pearson's product-moment correlation coefficient. IC_50_ was calculated using linear regression. Data was subjected to one-way ANOVA using SPSS software, 2011 version (*P* < 0.05).

## 3. Results and Discussion

### 3.1. Phytochemical Composition

It is well known that oxidation of biological substrates leads to various diseases. Antioxidants and phytochemicals from medicinal plants are now being used to counteract oxidation at the biological level, which is a safer approach than using pharmaceutical drugs to reduce oxidation.

The plants investigated are used in traditional medicine for various medicinal and pharmacological effects [14–19]. Studies also report that they exhibit good antioxidant activity* in vitro* [13, 31] Table 1 depicts the phytochemical composition of the samples, where it was observed that CS and AnP were good sources of polyphenols, glutathione, *β*-carotene, total saponins, and steroidal saponins, whereas AP and CP were found to be good sources of flavonoids, alkaloids, *α*-tocopherol, vitamin C, and tannins.

### 3.2. Polyphenol and Flavonoid Content of the Extracts

The polyphenol and flavonoid content of the extracts are given in Table 1. In general, all the extracts of the samples were found to be fair sources ofpolyphenol and flavonoids; however, it was high in the extracts of AP. The polyphenol content in two extracts of AnP (AQ and ME) was higher than in CP and CS, while the polyphenols in 80 ME of CP and CS was higher than AnP. Similarly, the flavonoid content of AP was high in all the extracts, followed by AnP > CP > CS. It may be inferred that methanol and 80% methanol are solvents suitable for extraction of polyphenol and flavonoid in these samples. Similar trend was observed in earlier studies on* Morus indica* [32, 33] and* Moringa oleifera* [34].

### 3.3. Inhibition of Oxidation

Lipid peroxidation is a complex process wherein the initially formed lipid radicals are converted to TBARS [35]. The first event of lipid peroxidation is oxygen absorption [36]. In Fenton's reaction, the free radical produced is hydroxyl radical which is the most deleterious and reactive radical among the ROS with shortest half-life. The oxygen derived hydroxyl radicals in presence of transition metal ion Fe^2+^ causes the degradation to form malondialdehyde (MDA) which produces a pink chromogen with thiobarbituric acid. MDA produced due to oxidative damage of biological substrates is an indicator of free radical pathology [37]. In the present work, TBARS was used to evaluate the antioxidative activity of different medicinal plant extracts and inhibition of generation of hydroxyl radicals by Fenton reaction in two biological substrates, microsomes and RBC.

#### 3.3.1. Microsomes

**Figure 1:**
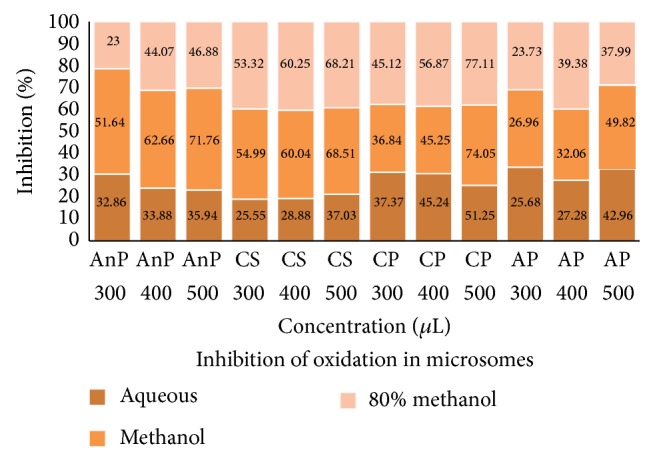
Inhibition of oxidation by different plant extracts in microsomes; AnP:* Andrographis paniculata*, CS:* Costus speciosus*, CP:* Canthium parviflorum*, and AP:* Abrus precatorius. *Values are the mean of triplicates (*n* = 3).

Microsomes due to their high PUFA content are susceptible to oxidation and hence are extensively used as a model for studying oxidative stress and antioxidant studies. The inhibition of oxidation at different concentrations (300, 400, and 500 *μ*L) by different solvent extracts of the sample is given in Figure 1. The activity was found to be dose dependent in all the extracts. At higher concentration, the inhibition was greater in 80 ME (77%) and ME (74%) of CP, CS (ME, 68% and 80 ME, 68%), and AnP (ME, 71%). In AQ extract the activity was low in all the samples and maximum percent inhibition observed was 51 ± 2.90% in CP. The results indicate that CP and CS were highly effective in inhibiting oxidation. The results are in accordance with an earlier study in* Morus indica* where ME and 80 ME extracts were potent in inhibiting the oxidation in microsomes [33].

#### 3.3.2. RBC

**Figure 2:**
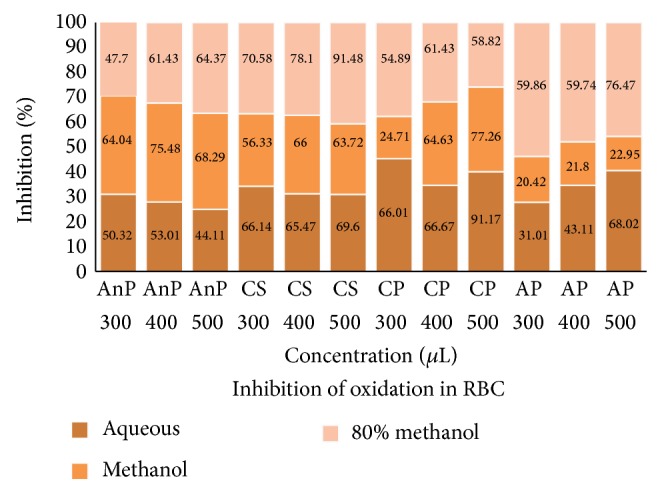
Inhibition of oxidation by different plant extracts in RBC. AnP:* Andrographis paniculata*, CS:* Costus speciosus*, CP:* Canthium parviflorum*, and AP:* Abrus precatorius.* Values are the mean of triplicates (*n* = 3).

Erythrocytes have been extensively used as a simple cell model to study oxidative stress. The inhibition of oxidation at different concentrations (300, 400, and 500 *μ*L) by different solvent extracts of the sample is given in Figure 2. The activity was found to be dose dependent in all the extracts of AP, whereas in the ME of CS and AnP, 80 ME of CP, and AQ of AnP the maximum activity was exhibited at 400 *μ*L; however no notable difference was observed in the activity between 400 and 500 *μ*L. Among all the extracts of the samples, maximum activity of more than 90% was shown by 80 ME of CS and AQ of CP.

As expected, TBARS values were effectively reduced by the plant extracts in biological substrates. This indicated that the extracts could efficiently prevent the nonenzymatic induced lipid peroxidation in various models [38]. The mode of action is primarily reducing oxidative damage by trapping the free radicals directly or scavenging them through a series of coupled reactions with antioxidant enzymes, contributing to stabilization of lipid peroxides [39].

Protective effect of* Ugni molinae* against oxidative damage of human erythrocytes is reported. HPLC-MS analysis of* Ugni molinae* leaves revealed the presence of polyphenols, flavonols and flavanols, myricetin, quercetin, and epicatechin [40]. In another study, the stem bark extract of* Semecarpus anacardium* showed significant protection against Fenton reaction induced lipid peroxidation in sheep liver tissue homogenate model and heat induced hemolysis in human RBC membrane model [37]. Hence, the antioxidant activity of these medicinal plants can be related to their phytochemical constituents.

### 3.4. IC_50_ of the Plant Extracts

**Figure 3:**
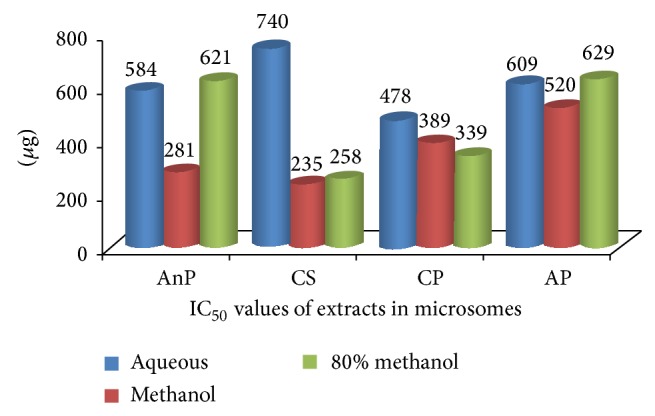
IC_50_ values of different solvent extracts of selected medicinal plants in microsomes; AnP:* Andrographis paniculata*, CS:* Costus speciosus*, CP:* Canthium parviflorum*, and AP:* Abrus precatorius.*

**Figure 4:**
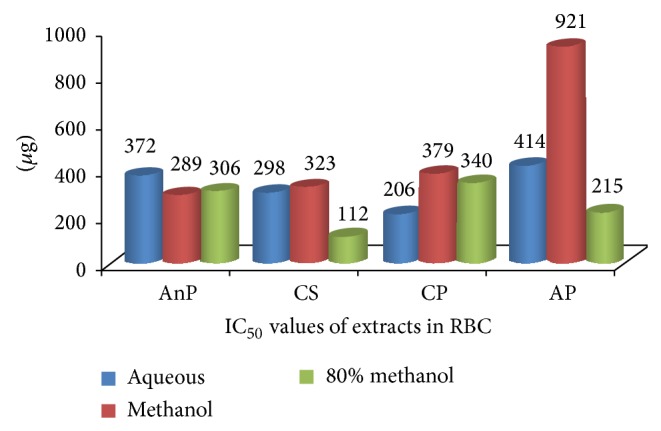
IC_50_ values of different solvent extracts of selected medicinal plants in RBC. AnP:* Andrographis paniculata*, CS:* Costus speciosus*, CP:* Canthium parviflorum*, and AP:* Abrus precatorius.*

Linear regression was used to calculate IC_50_ values. The IC_50_ values of different solvent extracts of the samples are depicted in Figure 3 (microsomes) and Figure 4 (RBC). In microsomes, it can be observed that the IC_50_ values of ME (235 *μ*g) and 80 ME of CS were lower than other extracts followed by ME of AnP and 80 ME of CP. In RBC, the IC_50_ value of 80 ME of CS (112 *μ*g) and, AP and AQ of CP was lower than other extracts. In rest of the extracts, no remarkable difference was observed; however, a high IC_50_ value (921 *μ*g) was observed in ME of AP.

### 3.5. Correlation between the Inhibition of Oxidation and Polyphenol and Flavonoids

The correlation was measured in all the extracts in both substrates (given in Tables 2 and 3). ME (*r* = 0.95; *P* ≤ 0.01) and 80 ME (*r* = 0.96; *P* ≤ 0.01) of CP and CS and 80 ME (*r* = 0.93; *P* ≤ 0.01) have shown a high correlation with both polyphenols and flavonoids indicating the protective effect of these phytochemicals on biological substrates. The inhibitory action might also be due to the presence of other phytochemicals such as glutathione, ascorbic acid. Literature reports the presence of alkaloids in these plants such as 2-amino-4-benzylthiomethyl-6-morpholino-1,3,5-triazine present in* Abrus precatorius* [41], the lactone andrographolide and flavonoids, 5,7,2′,3′-tetramethoxyflavanone and 5-hydroxy-7,2′,3′-trimethoxyflavone in* Andrographis paniculata* [42], flavonoids and phenolic compounds present in* Canthium parviflorum* [43], and alkaloids and flavonoids in* Costus speciosus* [44] which also might have imposed a protective effect.

## 4. Conclusion

It can be inferred from the findings that* Andrographis paniculata*,* Abrus precatorius*,* Canthium parviflorum*, and* Costus speciosus* possess ability to inhibit oxidation of biological substrates, by virtue of the presence of flavonoid and polyphenols. It was also observed that the extractability depends primarily on the solvent systems used. Further studies to isolate and identify the bioactive constituents are in progress for their optimal utilization.

## Figures and Tables

**Table 1:** Phytochemical composition of selected medicinal plants.

Phytochemicals	AnP	CS	CP	AP
Polyphenols^*^	3.21 ± 0.10^b^	3.21 ± 0.12^b^	1.28 ± 0.32^a^	1.06 ± 0.06^a^
Flavonoids^**^	1.50 ± 0.16^a^	1.17 ± 0.42^a^	2.44 ± 0.81^b^	1.32 ± 0.08^a^
Glutathione^***^	824 ± 28.84^c^	285.6 ± 33^a^	311.55 ± 25^ab^	375 ± 76.77^b^
*α*-Tocopherol^**^	NA	90.9 ± 3.29^b^	164.6 ± 22.10^c^	36.6 ± 3.05^a^
Tannins^**^	846 ± 0.15^b^	1088 ± 0.03^c^	605 ± 0.02^a^	1160 ± 0.07^c^
*β*-Carotene^****^	ND	1377 ± 2.51^b^	1060 ± 0.6^a^	1260 ± 0.05^b^
Alkaloids^**^	44.6 ± 6.65^a^	120 ± 0.03^b^	400 ± 0.06^c^	1100 ± 0.03^d^
Total sap.^**^	0.300^c^	0.282 ± 0.04^bc^	0.200 ± 0.04^a^	0.244 ± 0.01^ab^
Steroidal sap.^**^	0.110^a^	0.238 ± 0.038^c^	0.148 ± 0.051^ab^	0.186 ± 0.028^b^
Vitamin C^*^	ND	0.0237 ± 0.00^a^	0.0228 ± 0.00^a^	0.643 ± 0.04^b^

AnP: *Andrographis paniculata*, CS: *Costus speciosus*, CP: *Canthium parviflorum*, and AP: *Abrus precatorius*; ^*^g/100 g, ^**^mg/100 g, and ^***^mmol/100 g, ^****^ *μ*g/100 g dry basis. ND: not detected, sap.: saponins, and NA: not analyzed. Mean values with different superscripts a, b, c and d in rows, differ significantly (*P* ≤ 0.05).

**Table 2:** Relationship (*r* value) with the antioxidant effect and polyphenol and flavonoid content of plant extracts in microsomes.

	AQ	*r* value	ME	*r* value	80ME	*r* value
Polyphenol (mg/g)						
AnP	63.33 ± 4.71	0.233	88.33 ± 2.35	0.889	41.66 ± 2.35	0.532
CP	25.00 ± 0	0.792	46.66 ± 4.71	0.959	55.00 ± 0	0.964
CS	55.00 ± 0	0.706	51.66 ± 2.35	0.716	63.33 ± 2.35	0.915
AP	81.66 ± 8.49	0.828	83.33 ± 9.42	0.951	120.00 ± 0	0.786
Flavonoid (mg/g)						
AnP	0.59 ± 0.02	0.232	1.87 ± 0.07	0.889	0.96 ± 0.11	0.532
CP	0.01 ± 0	0.790	0.59 ± 0.01	0.941	1.09 ± 0.01	0.957
CS	0.16 ± 0.03	0.669	0.04 ± 0	0.710	0.40 ± 0.06	0.914
AP	1.02 ± 0.08	0.828	2.58 ± 0.10	0.943	1.69 ± 0.02	0.786

Aq: aqueous extract; ME: methanol extract; 80ME: 80% methanol extract;

AnP: *Andrographis paniculata*, CS: *Costus speciosus*, CP: *Canthium parviflorum*, and AP: *Abrus precatorius*. Values are the mean of triplicates (*n* = 3).

**Table 3:** Relationship (*r* value) with the antioxidant effect and polyphenol and flavonoid content of plant extracts in RBC.

	AQ	*r* value	ME	*r* value	80ME	*r* value
Polyphenol (mg/g)						
AnP	63.33 ± 4.71	−0.578	88.33 ± 2.35	0.270	41.66 ± 2.35	0.900
CP	25.00 ± 0	0.725	46.66 ± 4.71	0.882	55.00 ± 0	0.366
CS	55.00 ± 0	0.235	51.66 ± 2.35	0.584	63.33 ± 2.35	0.937
AP	81.66 ± 8.49	0.970	83.33 ± 9.42	0.455	120.00 ± 0	0.790
Flavonoid (mg/g)						
AnP	0.59 ± 0.02	−0.55	1.87 ± 0.07	0.270	0.96 ± 0.11	0.900
CP	0.01 ± 0	0.779	0.59 ± 0.01	0.901	1.09 ± 0.01	0.394
CS	0.16 ± 0.03	0.227	0.04 ± 0	0.659	0.40 ± 0.06	0.931
AP	1.02 ± 0.08	0.970	2.58 ± 0.10	0.455	1.69 ± 0.02	0.790

Aq: aqueous extract; ME: methanol extract; 80ME: 80% methanol extract.

AnP: *Andrographis paniculata*, CS: *Costus speciosus*, CP: *Canthium parviflorum*, and AP: *Abrus precatorius*. Values are the mean of triplicates (*n* = 3).
